# Pilot tone navigation for respiratory and cardiac motion‐resolved free‐running 5D flow MRI

**DOI:** 10.1002/mrm.29023

**Published:** 2021-10-05

**Authors:** Mariana B. L. Falcão, Lorenzo Di Sopra, Liliana Ma, Mario Bacher, Jérôme Yerly, Peter Speier, Tobias Rutz, Milan Prša, Michael Markl, Matthias Stuber, Christopher W. Roy

**Affiliations:** ^1^ Department of Diagnostic and Interventional Radiology Lausanne University Hospital (CHUV) and University of Lausanne (UNIL) Lausanne Switzerland; ^2^ Department of Radiology Feinberg School of Medicine Northwestern University Chicago Illinois USA; ^3^ Department of Biomedical Engineering Northwestern University Chicago Illinois USA; ^4^ Siemens Healthcare GmbH Erlangen Germany; ^5^ Advanced Clinical Imaging Technology Siemens Healthcare AG Lausanne Switzerland; ^6^ Center for Biomedical Imaging (CIBM) Lausanne Switzerland; ^7^ Service of Cardiology Centre de Resonance Magnétique Cardiaque (CRMC) Lausanne University Hospital and University of Lausanne Lausanne Switzerland; ^8^ Woman‐Mother‐Child Department Lausanne University Hospital and University of Lausanne Lausanne Switzerland

**Keywords:** 5D flow MRI, cardiac motion, free‐running, pilot tone, respiratory motion

## Abstract

**Purpose:**

In this work, we integrated the pilot tone (PT) navigation system into a reconstruction framework for respiratory and cardiac motion‐resolved 5D flow. We tested the hypotheses that PT would provide equivalent respiratory curves, cardiac triggers, and corresponding flow measurements to a previously established self‐gating (SG) technique while being independent from changes to the acquisition parameters.

**Methods:**

Fifteen volunteers and 9 patients were scanned with a free‐running 5D flow sequence, with PT integrated. Respiratory curves and cardiac triggers from PT and SG were compared across all subjects. Flow measurements from 5D flow reconstructions using both PT and SG were compared to each other and to a reference electrocardiogram‐gated and respiratory triggered 4D flow acquisition. Radial trajectories with variable readouts per interleave were also tested in 1 subject to compare cardiac trigger quality between PT and SG.

**Results:**

The correlation between PT and SG respiratory curves were 0.95 ± 0.06 for volunteers and 0.95 ± 0.04 for patients. Heartbeat duration measurements in volunteers and patients showed a bias to electrocardiogram measurements of, respectively, 0.16 ± 64.94 ms and 0.01 ± 39.29 ms for PT versus electrocardiogram and of 0.24 ± 63.68 ms and 0.09 ± 32.79 ms for SG versus electrocardiogram. No significant differences were reported for the flow measurements between 5D flow PT and from 5D flow SG. A decrease in the cardiac triggering quality of SG was observed for increasing readouts per interleave, whereas PT quality remained constant.

**Conclusion:**

PT has been successfully integrated in 5D flow MRI and has shown equivalent results to the previously described 5D flow SG technique, while being completely acquisition‐independent.

## 1 INTRODUCTION

4D flow MRI provides a quantitative evaluation of hemodynamics across an entire 3D volume, allowing for simultaneous assessment of flow in multiple vessels and cardiac chambers.^1^, ^2^ As a result, 4D flow has become an integral part of the diagnosis and patient management for disorders such as congenital heart disease (CHD) and valvar abnormalities.^3^, ^4^ Typically, diaphragmatic navigators are used to monitor respiratory displacement and discard data acquired during inspiration, thus eliminating blurring artifacts caused by respiratory motion. However, the efficiency of these respiratory navigators depends on the patient’s physiology and anatomy, causing unpredictable scan times.^5^ Consequently, it becomes challenging to routinely acquire 4D flow datasets covering the heart and great vessels (whole‐heart coverage) in a clinically acceptable time (<10 min).

To improve scanning efficiency, several methods have been proposed to collect flow data throughout the entire respiratory cycle and either retrospectively trigger,^6^ correct,^7^ or resolve respiratory motion.^8^, ^9^, ^10^, ^11^ Here, as in other studies, we refer to respiratory and cardiac motion‐resolved volumetric flow imaging as 5D flow imaging.

Existing approaches for 5D flow imaging take advantage of self‐gating (SG), where physiological motion can be directly derived from the acquired imaging data. To resolve respiratory motion, the datasets are binned according to the amplitude of a SG respiratory curve, whereas cardiac motion is resolved by binning data according to time points derived from SG cardiac triggers, effectively removing the need for electrocardiography (ECG) placement and thereby promoting a faster and simpler patient setup.^8^, ^9^, ^10^, ^11^

The main drawback behind SG strategies is their dependence on the periodic sampling of either a point or a 1D readout,^6^, ^7^, ^8^, ^9^, ^10^, ^11^ which if not sampled frequently enough may limit the precision of the SG respiratory curves and, especially, cardiac triggers. Likewise, for the SG strategies requiring the repetition of 1D readouts, the limitations of gradient hardware and the need to minimize both eddy current effects and sequence dependent artifacts^12^, ^13^ limit our ability to arbitrarily switch between imaging and SG readouts without impacting scanning efficiency and the final image quality. This issue is further confounded by flow sequences, which repeat each readout multiple times for velocity encoding. It would therefore be of interest to find a reliable alternative to extract respiratory curves and cardiac triggers with high sampling rate without impacting the image acquisition scheme.

Recently, the pilot tone (PT) navigation system was proposed as an MR image‐independent motion detection system.^14^ The PT navigation system, implemented by Speier et al.,^14^, ^15^, ^16^ consists of a small loop antenna, integrated inside a chest coil array, that transmits a continuous‐wave RF signal into the magnet bore at a frequency outside of the frequency band of the MR imaging signal, ergo not disturbing the image acquisition but still inside the useable receiver bandwidth. This signal is then captured by all active receiver coils after having been modulated by the underlying motion. From this signal, it is possible to extract respiratory curves in agreement with conventional MR navigators,^14^, ^17^ as well as cardiac triggers comparable to ECG gating,^15^, ^16^ all in parallel to the MRI acquisition. Thus, PT may be a valuable alternative to the aforementioned MR data‐driven SG approaches by providing signals with a higher sampling rate that are independent from the image acquisition.

The goal of this work was the integration of the PT navigation system into a recently proposed free‐running radial flow framework for respiratory‐ and cardiac motion‐resolved radial 5D flow imaging.^9^, ^18^ PT was compared to the previously described SG method and was validated in the 5D flow framework for healthy subjects and patients with CHD. As a reference measurement, 5D flow reconstructions were additionally compared to conventional ECG‐triggered and respiratory navigated Cartesian 4D flow acquisitions. We tested the following 3 hypotheses: 1) PT provides equivalent respiratory curves and cardiac triggers to SG as part of a published 5D flow protocol; 2) 5D flow image reconstruction using PT yields equivalent flow measurements with respect to 5D flow reconstructions of the same data using SG; 3) PT signals, unlike SG, are unaffected by changes to the underlying 3D radial sequence trajectory.

## 2 METHODS

### 2.1 Study cohort and data acquisition

A cohort of 15 healthy adults (7 female, age 23‐34 years) and 9 patients (3 female, age 13‐55 years) with CHD (pathologies listed in Supporting Information Table S1) were scanned on a 1.5T Magnetom Sola (Siemens Healthcare, Erlangen, Germany) using a 12‐channel body coil array with an integrated PT generator. All subjects participating in this study, or their legal guardians in case of minors, provided written informed consent compliant with our institutional guidelines and approved by the local research ethics committee.

For each subject, a prototype free‐running radial 3D whole‐heart flow sequence — hereafter referred to as *5D flow sequence* ^9^ — was acquired, and PT data was recorded with every readout of the flow sequence by activating the system’s integrated PT signal detection functionality. For reference, a conventional ECG gated respiratory navigated Cartesian 4D flow sequence covering the aorta was also acquired.^5^ Scan parameters are provided in Table 1.

**TABLE 1:** Scan parameters for 5D flow and reference 4D flow acquisitions

	5D flow		4D flow reference	
Trajectory	3D radial		3D Cartesian	
Respiration	Gated		Triggered	
Cardiac gating	ECG/SG/PT		ECG	
TE/TR	2.93/4.67 ms		2.33/5.08 ms	
RF excitation angle	7º		7º	
Coverage	Whole heart		Aortic arch	
Acquisition efficiency	100%		32– 97%	
Acceleration rate	R = 43‐75		GRAPPA, R = 2	
	**Healthy cohort**	**Patient cohort**	**Healthy cohort**	**Patient cohort**
Venc	150 cm/s	150‐200 cm/s	150 cm/s	150 cm/s
Temporal resolution	38.3‐40 ms	38.5‐40 ms	21.6‐38.1 ms	39.9‐40.8 ms
Spatial resolution	2.5 × 2.5 × 2.5 mm^3^	[2.1‐2.5] × [2.1‐2.5] × [2.1‐2.5] mm^3^	2.5 × 2.5 × 2.5 mm^3^	2.5 × 2.5 × 2.5 mm^3^
FOV	240 × 240 × 240 mm^3^	(200‐240) × (200‐240) × (200‐240) mm^3^	(200‐300) × (360‐420) × (75‐90) mm^3^	(166.7‐240) × (300‐360) × (83.2‐110) mm^3^
Acquisition time	7:53 min	7:53‐8:55 min	4:25‐12:34 min	4:42‐9:29 min

Scan time in CHD patients was adapted for each clinical case, depending on resolution, FOV, and maximum venc.

CHD, congenital heart disease; ECG, electrocardiogram; PT, pilot tone; SG, self‐gating; venc, velocity encoding.

### 2.2 5D flow pulse sequence

The 5D flow framework implemented in the present study^9^ is based on a previously reported free‐running framework for 5D radial whole‐heart imaging^18^ that continuously samples k‐space following a 3D radial spiral phyllotaxis sampling pattern.^19^ In the 5D flow setup, several spiral interleaves are acquired sequentially and rotated by the golden angle. Each interleave includes a readout orientated along the superior‐inferior (SI) direction for subsequent extraction of SG respiratory curves and cardiac triggers,^18^ followed by a series of radial imaging readouts spiraling down k‐space. Every imaging readout, aside from the SI readouts, was repeated 4 times for balanced 4‐point velocity encoding. In order to ensure a sufficient sampling rate of the SI projections for extracting cardiac motion, the number of radial angles sampled per interleave (excluding SI readouts) was established as 5, resulting in a total of 21 readouts per interleave (1 SI + (5 readouts × 4 velocity encoding)). The described 5D flow framework has been previously validated both in vitro and in vivo in a cohort of patients with aortic disease.^9^ In addition to using SI readouts for SG (sampling frequency of 10.2 Hz), PT signals were extracted at every readout in parallel to the image acquisition (sampling frequency of 214.1 Hz), and gold standard ECG signals (sampling frequency of 400 Hz) were also recorded throughout the 5D flow scan for subsequent cardiac triggering comparisons.^18^

### 2.3 Physiological signal extraction

**FIGURE 1:**
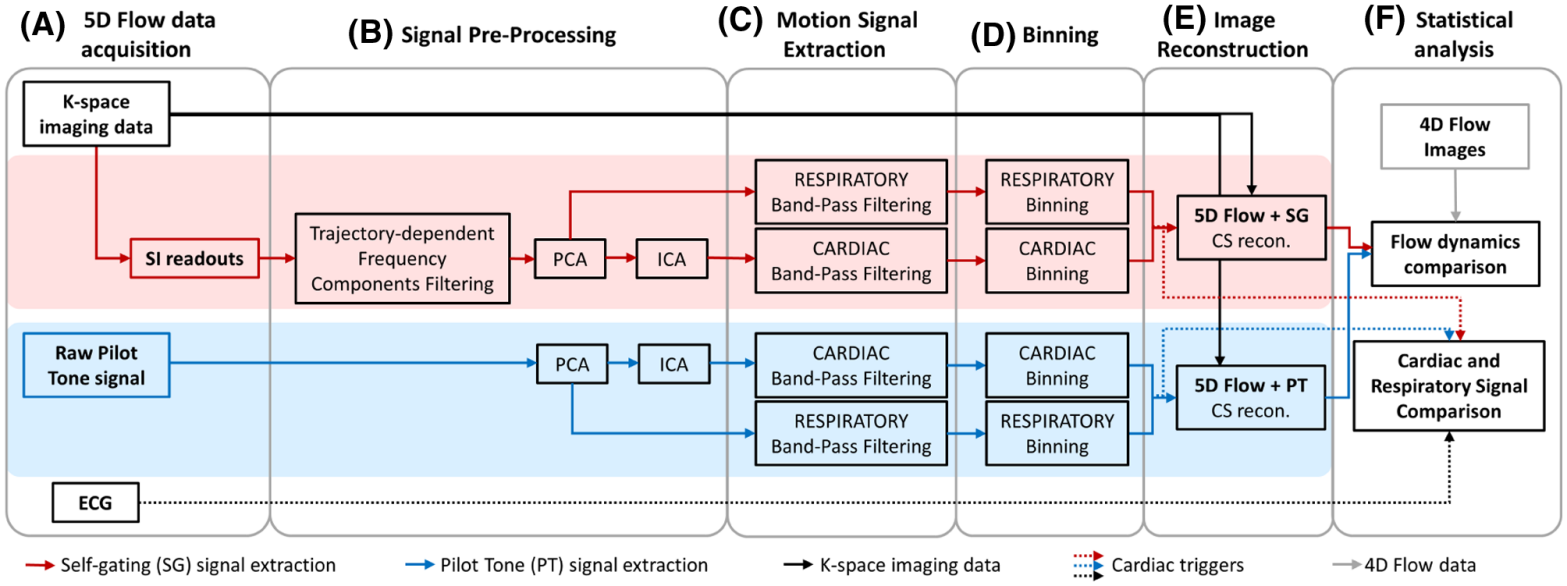
Schematic of the pipeline used for this work. (A) The acquired 5D flow datasets were used to extract SG respiratory curves and cardiac triggers. In parallel, PT signals were also used to extract respiratory curves and cardiac triggers. (B) SG signals were corrected for trajectory dependent artifacts. Both SG and PT signals were preprocessed using principal component analysis and independent component analysis. (C) The range of respiratory and cardiac motion was used to find the best representation of each modulation. The extracted respiratory curves from SG and PT were compared with each other, and cardiac triggers were compared with each other as well as with ECG triggers (F). (D) Finally, the 5D flow dataset was binned into respiratory and cardiac phases based on the PT signals (5D flow PT), SG signals (5D flow SG), and (E). XD‐GRASP was used to reconstruct both datasets. (F) Finally, flow hemodynamics of the 5D flow PT and 5D flow SG were compared with conventional 4D flow. ECG, electrocardiogram; PT, pilot tone; SG, self‐gating

The approach for both PT and SG signal extraction frameworks was based on the work by Di Sopra et al.^18^ for the free‐running 5D framework, which is summarized in Figure 1. This signal extraction pipeline was implemented in MatLab R2018b (MathWorks, Natick, MA). First, for SG, the SI readouts extracted from the imaging data were corrected for trajectory‐dependent imperfections caused by eddy currents, gradient timing delays, and the magnetohydrodynamic effect. The effect of these trajectory imperfections in the SG signal is related to the chosen trajectory architecture for image acquisition.^18^ Conversely, the PT signals, emitted at a different frequency, are not affected by the same confounding effects in the current setup; thus, no correction algorithm was performed on those signals. The frequency spectrum for both SG and PT signals in 1 representative subject, before (PT and SG) and after (only SG) correcting for the trajectory imperfections, is shown in Supporting Information Figure S1.

The remaining signal extraction steps were identical for both PT and SG pipelines. Principal component analysis was applied to reduce data complexity and to segregate the respiratory and cardiac components. From the first ten principal components, an estimate of the subject‐specific frequency range of the respiratory motion was retrieved, and the principal component with the strongest modulation over the identified range was selected to describe the respiratory curve. To extract cardiac motion, independent component analysis was performed on top of the previously extracted principal components. Pre‐applying principal component analysis to these high dimensional datasets reduces computational complexity and enhances independent component analysis performance.^20^, ^21^ Then, the subject‐specific frequency range of cardiac motion was estimated, and the independent component with the strongest modulation in the defined range was singled out to represent the cardiac signal. From the SG cardiac signals, similarly to the implementation by Di Sopra et al.,^18^ cardiac triggers were marked at the zero‐crossing time points. Conversely, for PT gating, the local minima was chosen as the trigger point.^15^

### 2.4 Data sorting

For each signal extraction type (PT and SG), the individual readouts were assigned to bins independently of their position in the originally acquired interleave; however, to ensure consistency, velocity encodes for a given readout were assigned to the same respiratory and cardiac phase, creating 6D arrays (k_x_‐k_y_‐k_z_‐respiratory‐cardiac‐velocity encode). The resulting 5D flow datasets sorted using PT and using SG will be hereafter referred to as 5D flow PT and 5D flow SG, respectively. For both PT and SG, the extracted respiratory curve was used to partition the acquired 5D flow data into 4 equally distributed respiratory motion states, ranging from end‐inspiration to end‐expiration, according to the amplitude of signal at each time point. Similarly, data were also assigned to different cardiac phases using the extracted cardiac triggers. The width of each cardiac bin was fixed to 40 ms, resulting in a variable number between 19 and 31 cardiac phases obtained from binning, depending on the individual heart rate of each subject.

### 2.5 Image reconstruction

5D flow PT and 5D flow SG images were reconstructed offline using the previously described free‐running framework,^18^ wherein a multidimensional compressed sensing algorithm enforces sparsity along both respiratory and cardiac dimensions.^22^, ^23^ All SI projections, previously used for SG, were removed prior to image reconstruction. Respiratory and cardiac regularization weights were set to 0.005 and 0.0075, respectively, matching those used previously.^9^ All 5D flow reconstructions were performed using MatLab R2018b (MathWorks) on a workstation equipped with 2 Intel Xeon CPUs (Intel, Santa Clara, CA), 512GB of RAM, and a NVIDIA Tesla GPU (Nvidia, Santa Clara, CA). Reconstruction time for each 5D flow dataset varied from 8‐13 h, depending on the number of cardiac phases and the number of active receiver channels. Conversely, reference 4D flow image reconstructions were directly provided by the scanner reconstruction pipeline during the examination.

### 2.6 Analysis of respiratory curves and cardiac triggers

To test our first hypothesis, that PT provides equivalent physiological signals to SG, quantitative comparison of respiratory curves extracted using PT and SG was performed by measuring the consistency between data binned with PT and SG gating, respectively, defined by the percentage of coinciding (overlapping) data points between respiratory phases. Additionally, for each 5D flow PT and 5D flow SG reconstruction, the end‐expiration and end‐inspiration images were rigidly coregistered over a region of interest containing the lung–liver interface using NiftyRegv1.3.9Ad (University College London, United‐Kingdom).^24^, ^25^ The resulting displacement measure along the SI direction was used to quantitatively compare respiratory motion detection from the PT and SG signals.

Quantitative comparison of cardiac triggers was performed by comparing the time between consecutive triggers (heartbeat interval duration) derived from PT and SG to gold standard ECG. Additionally, the trigger jitter was defined and calculated as the standard deviation across the trigger delays between every pair of corresponding triggers for either PT or SG versus ECG.^13^ To exclude missed triggers from the analysis, rejection of individual triggers was performed using an outlier rejection strategy described previously.^18^ This strategy excludes heartbeat intervals that are 1.5 times longer or 0.5 times shorter than the median heartbeat estimated for 20 consecutive heartbeats around each interval.^18^ Two subjects had more than 1% of reported corrupted ECG cardiac triggers and were therefore excluded from the cardiac trigger comparison. For the remaining subjects, only individual ECG triggers were excluded.

### 2.7 Analysis of flow measurements

To test our second hypothesis, that PT enables equivalent flow measurements to SG, the images reconstructed from 5D flow and 4D flow datasets were first preprocessed using noise filters, background phase correction, and anti‐aliasing correction. A second‐order 3D background phase correction model was implemented for 5D flow imaging,^9^ whereas a first order correction was used for 4D flow acquisitions.^1^, ^2^ The order of each correction model differed because the phase offset is derived from eddy currents, and this offset depends on the type of trajectory used (Cartesian, radial, etc.).

For this analysis, only the end‐expiratory phase images of 5D flow PT and 5D flow SG were used because the focus of this study was not to understand the differences in respiratory hemodynamics but instead to validate PT as a valid alternative to SG for 5D flow imaging. The acquired 4D flow datasets were included in the analysis as a reference measurement. For each flow dataset included in this study, the time‐averaged phase‐contrast angiogram was calculated using the magnitude and phase images of each dataset. From these phase‐contrast angiogram images, a segment of the aorta was selected based on image thresholding.

Four aortic 2D planes were manually selected for our comparison. The first plane was located at the lower ascending aorta, slightly above the aortic root. The second plane was located at the upper ascending aorta, before the aortic arch. The third plane was located at the end of the aortic arch (Arch), and the final plane was located at the distal descending aorta (DAo) between the third plane and the diaphragm. The flow rate was computed for each 2D plane across the entire cardiac cycle. Net flow (flow volume across a cardiac cycle), peak flow rate, and peak velocity were calculated per slice for all 5D flow datasets, as well as for the control 4D flow datasets. All segmentations and measurements were computed using the Siemens 4D Flow v2.4 software (Siemens Healthcare, Erlangen, Germany).

### 2.8 Impact of sequence parameters on PT and SG signals

To test our third hypothesis, that PT signals are not impacted by acquisition parameters that otherwise affect SG signals, 6 back‐to‐back 5D flow acquisitions with a reduced scan time (2:03 min) were performed in 1 healthy subject. Each acquisition used a different phyllotaxis trajectory architecture by varying the number of readouts acquired per interleave but keeping the total number of readouts constant (Table 2). Each interleave included 1 SI readout for self‐gating and a remaining set of readouts (varying for each acquisition), repeated 4 times for velocity encoding. Increasing the number of readouts acquired per interleave has the effect of decreasing the gradient strength required to move through k‐space but also lowers the sampling rate of SG signals. To assess the impact of eddy‐currents induced in each acquisition, the mean background velocity contained in manually selected static structures near the heart was measured. Additionally, PT and SG cardiac triggers were extracted; the sampling frequency was calculated; and the heartbeat interval and trigger jitters were computed using ECG as a reference. Note that this analysis was performed after an upgrade of our MRI system, which improved the PT sampling rate (2000 Hz).

**TABLE 2:** Impact of sequence parameters on PT and SG signals

Nread	ECG Heartbeat duration (ms)	SG Heartbeat Duration (ms)	PT Heartbeat Duration (ms)	SG Trigger Jitter (ms)	PT Trigger Jitter (ms)	SG Sampling Frequency (Hz)	PT Sampling Frequency (Hz)	Average Background Velocity (cm/s)
13	983 ± 68	981 ± 65	981 ± 72	10.6	19.2	16.4	2000	1.10
21	993 ± 77	994 ± 67	994 ± 72	12.4	9.9	10.2	2000	0.62
45	962 ± 69	954 ± 93	962 ± 67	20.6	11.9	4.7	2000	0.46
69	883 ± 58	890 ± 113	883 ± 58	60.1	9.8	3.1	2000	0.17
93	956 ± 48	960 ± 81	957 ± 39	109.9	12.6	2.3	2000	0.15
189	915 ± 54	2330 ± 99	914 ± 51	‐	14.7	1.1	2000	0.08

Six back‐to‐back 5D flow acquisitions with a variable Nread are shown. Overall, increasing Nread leads to decreased background velocity error but also decreased SG sampling rate and accuracy, precluding identification of cardiac triggers for high values of Nread. Conversely, PT sampling rate and accuracy remains unaffected by the underlying k‐space trajectory.

Nread, number of readouts acquired per interleave.

### 2.9 Statistical analysis

Agreement between PT and SG respiratory curves was assessed in healthy subjects and CHD patients by measuring the Pearson correlation coefficient. Quantitative measurements of liver displacement from 5D flow PT and 5D flow SG images were statistically compared using a paired *t* test.

Heartbeat intervals from PT and SG were compared to the corresponding ECG heartbeat intervals, which were automatically estimated throughout the scan (PT vs. ECG and SG vs. ECG) across all subjects using Bland‐Altman plots. From those Bland‐Altman plots, the mean and SD of the bias between each 2 modalities were reported. The cardiac trigger jitter measurements were compared between PT versus ECG and SG versus ECG using a paired *t* test.

In order to compare flow measurements between 5D flow PT and 5D flow SG, we calculated the net flow, peak flow rate, and peak velocity in the 5D flow PT datasets at each plane (aortic root, aortic arch, Arch, DAo) and by comparing these measurements to the 5D flow SG datasets as well as to the reference 4D flow using a paired *t* test between every 2 datasets (5D flow PT vs. 5D flow SG, 5D flow PT vs. 4D flow, and 5D flow SG vs. 4D flow). Bonferroni correction was performed to compensate for the 3 flow dataset comparisons. Finally, net flow and peak flow rate were compared for bias between each 2 datasets for all planes using Bland‐Altman plots.

## 3 RESULTS

### 3.1 Analysis of respiratory curves

The respiratory curve analysis from the healthy cohort (Table 3) revealed some small differences in binning when using PT versus SG. The average percentage of overlapping respiratory bins between the 2 modalities was 84.2% in end‐expiration, 75.1% and 81.9% in the 2 mid‐respiratory phases, and 90.7% in end‐inspiration. Results from the patient cohort revealed similar (albeit lower) agreement between PT and SG respiratory bins (Table 3), with an average overlapping percentage of 80.8% for end‐expiration 69.3% and 74.9% in the 2 mid‐respiratory phases and 86.9% for end‐inspiration. Binning mismatch between PT and SG was mostly distributed across the neighboring bins with a distribution between 8.6% and 18.7%.

**TABLE 3:** Respiratory binning consistency assessment between SG and PT datasets across all 15 healthy subjects and 9 CHD patients

		PT Binning							
		Healthy Subjects				CHD Patients			
		End‐exp	Mid‐exp	Mid‐insp	End‐insp	End‐exp	Mid‐exp	Mid‐insp	End‐insp
SG binning	End‐exp	**84.2 %**	15.1 %	0.5 %	0.2 %	**80.8 %**	18.7 %	0.4 %	0.3 %
	Mid‐exp	15.6 %	**75**.**1** %	8.8 %	0.5 %	18.6 %	**69.3 %**	11.9 %	0.2 %
	Mid‐insp	0.1 %	9.3 %	**81**.**9** %	8.6 %	0.5 %	11.7 %	**74.9 %**	12.8 %
	End‐insp	0.03 %	0.5 %	8.8 %	**90**.**7** %	0 .01%	0 .3%	12.8 %	**86.9 %**

The percentage of overlapping data points was measured for every pair of PT and SG bins. In general, non‐overlapped bins from the same respiratory phase were assigned to the neighboring phases.

**FIGURE 2:**
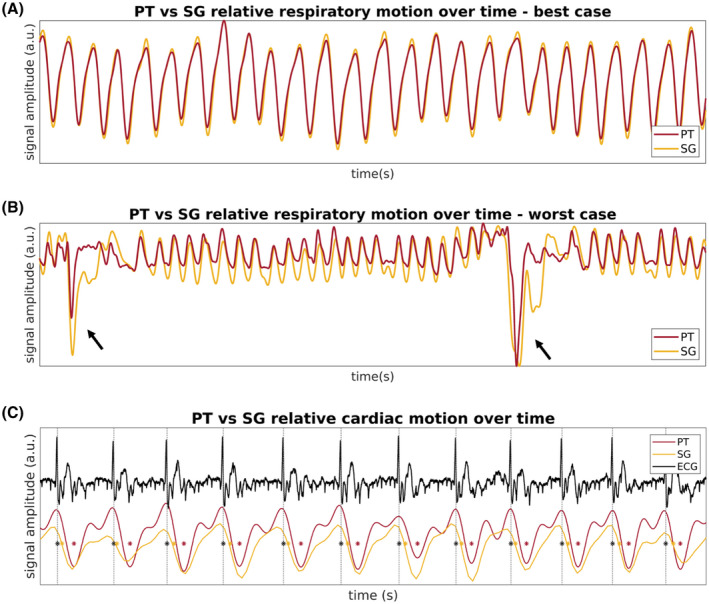
Respiratory and cardiac signals extracted from PT and SG plotted for a set of representative subjects. (A) Representation of the PT and SG relative respiratory curves over time for the healthy subject who had the highest reported Pearson correlation coefficient between PT and SG signals (0.99). (B) PT and SG relative respiratory curves over time for the healthy subject reporting the lowest Pearson correlation coefficient. Black arrows denote instances during the scan where the subject took deep breaths at irregular intervals (0.81). (C) Visualization of the PT and SG cardiac signals and their corresponding ECG signal for 1 representative subject demonstrating similar, albeit out‐of‐phase periodic detection of cardiac motion. Cardiac triggers for each modality are marked with colored asterisks (*)

The Pearson correlation coefficient between the extracted PT and SG respiratory curves was 0.95 ± 0.06 for healthy subjects and 0.95 ± 0.04 for patients. The PT and SG respiratory curves for a representative healthy subject reporting high signal correlation (0.99, *P* < .05) are shown in Figure 2A, B. depicts the PT and SG respiratory curves from the healthy subject reporting the lowest signal correlation (0.81, *P* < .05), where signal baseline drifts of different amplitude are found for both modalities.

The mean displacement of the liver measured between end‐expiratory and end‐expiratory 5D flow PT and 5D flow SG images were comparable in both the healthy volunteers (PT: 11.19 ± 3.66 mm, SG: 10.65 ± 3.81 mm, *P* = .57) and patient (PT: 9.45 ± 3.70 mm, SG: 9.14 ± 3.54 mm, *P* = .25) cohorts.

### 3.2 Analysis of cardiac triggers

**FIGURE 3:**
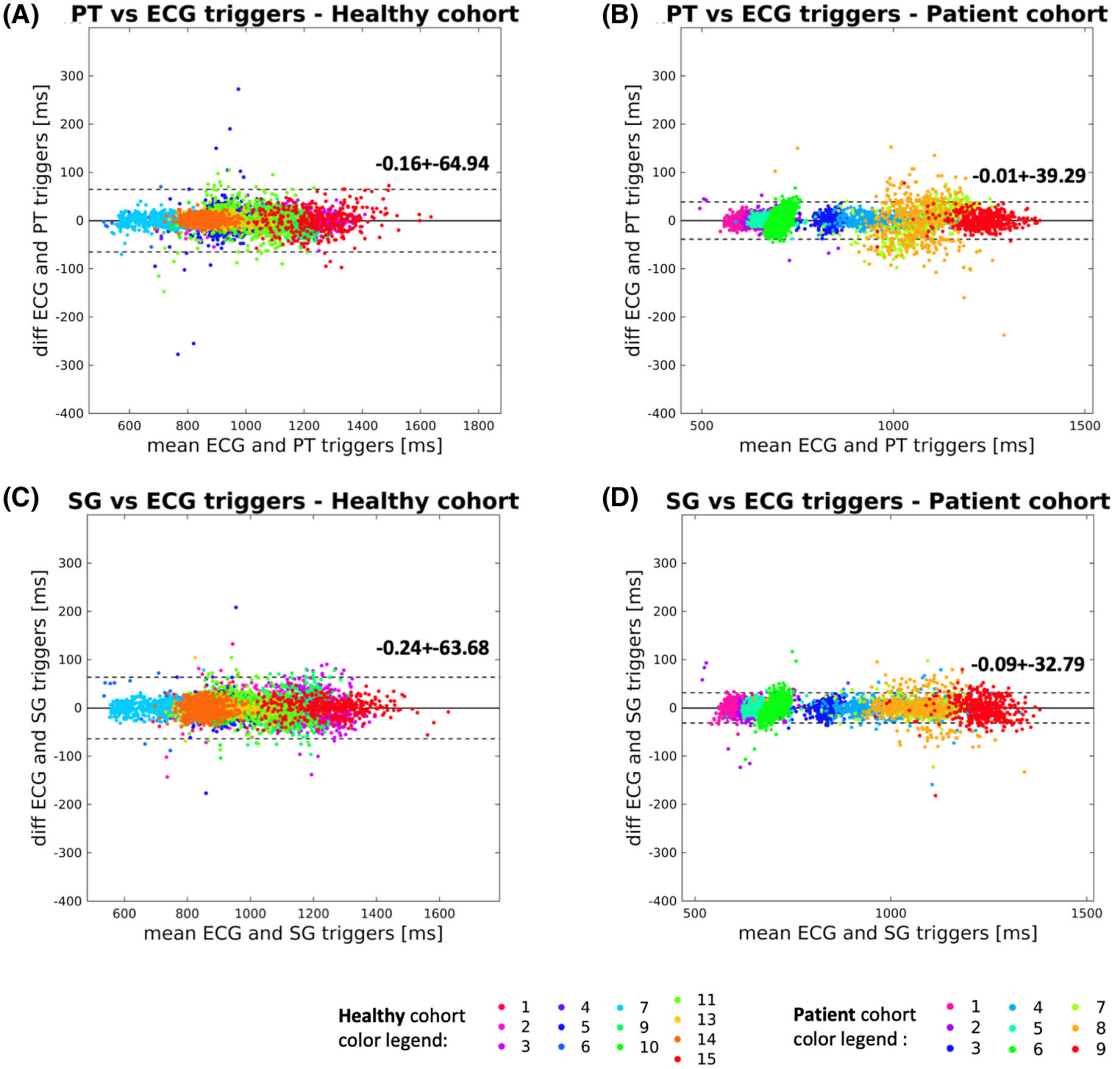
Quantitative comparison of heartbeat estimations from 13 healthy subjects (2 subjects were excluded from this analysis) and 9 patients. Each Bland –Altman plot compares differences between the ECG heartbeat interval duration for all cardiac intervals and the corresponding heartbeat duration estimated from PT (A,B) and SG (C,D) cardiac triggers. The linear correlation between the heartbeat durations in both healthy subjects (A,C) and patients (B,D) is excellent r^2^ > 0.95. The 2 healthy subjects excluded from this analysis had limited ECG quality (>1% of reported corrupted triggers)

One subject reported 4 missed ECG triggers, which were excluded from the remainder of the analysis. Additionally, 1 subject reported 3 missed PT triggers, and another subject reported 2 missed SG triggers. Overall, both PT and SG heartbeat interval duration measures showed a low bias with ECG (Figure 3). Bias values for each Bland‐Altman plot were 0.16 ± 64.94 ms for PT versus ECG and 0.24 ± 63.68 ms for SG versus ECG in healthy subjects, and were 0.01 ± 39.29 ms for PT versus ECG and 0.09 ± 32.79 ms for SG versus ECG in patients. Figure 2C shows 3 cardiac signals (PT, SG, and ECG) together with their respective triggers extracted for 1 representative subject during a 5D flow acquisition.

Trigger jitter measurements did not show significant differences (*P* > .05) between PT versus ECG and SG versus ECG. Values reported in healthy subjects were 13.9 ± 8.2 ms for PT versus ECG and 17.0 ± 4.6 ms for SG versus ECG. These values correspond to 1.4 ± 0.7 % and 1.7 ± 0.4 % of the average heartbeat duration, respectively. The same measurements in patients reflected similar results, being 13.0 ± 5.7 ms for PT versus ECG and 17.3 ± 4.9 ms for SG versus ECG, or equivalently representing 1.3 ± 0.6% and 1.7 ± 0.4% of the average heartbeat duration.

### 3.3 Analysis of flow measurements

**FIGURE 4:**
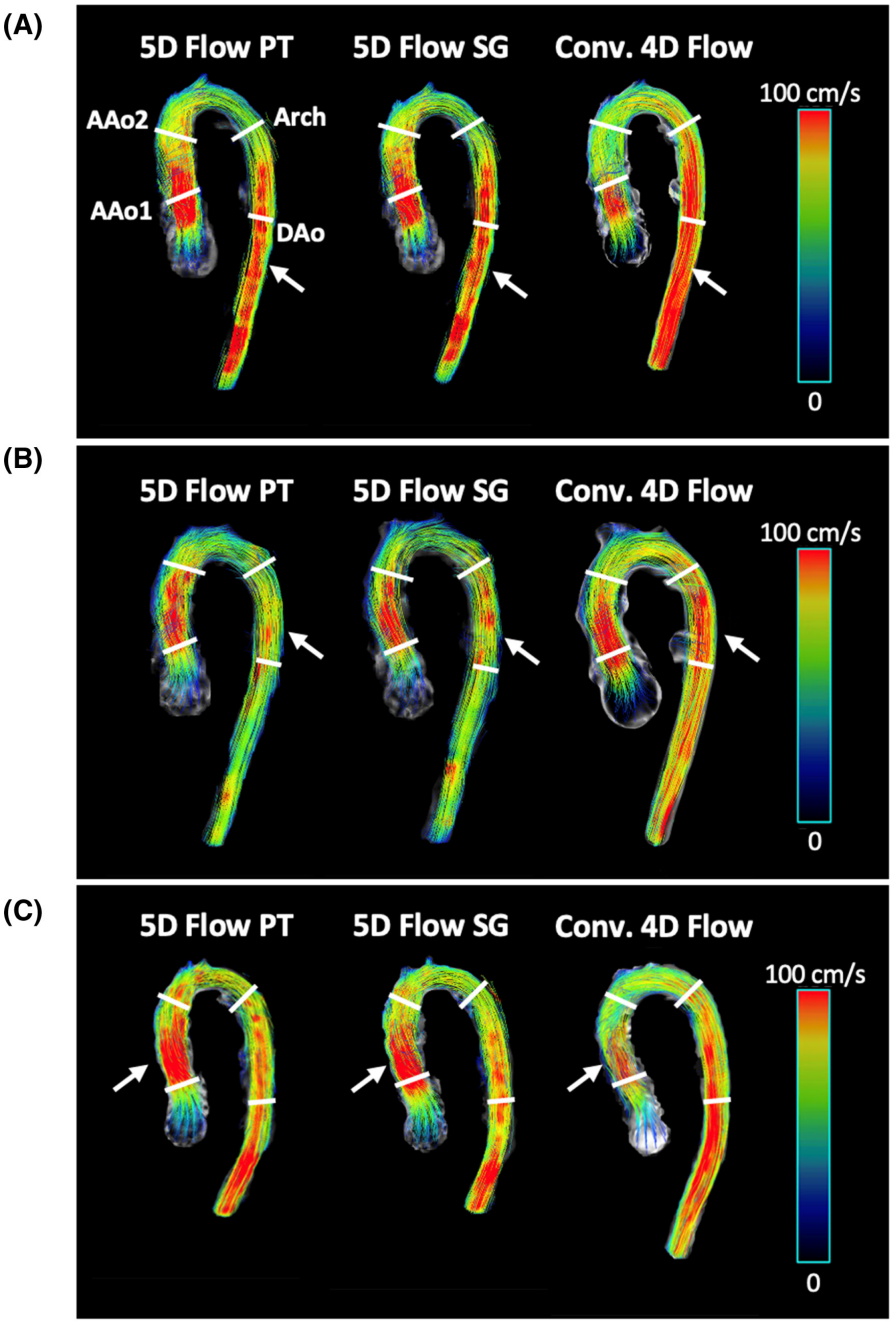
Flow streamlines in the aorta. Flow streamlines are displayed for 2 representative healthy volunteers (A‐B) and 1 representative patient (C). Four 2D segments (ascending aorta: AAo1, ascending aorta pre‐aortic arch: AAo2, end of aortic arch: Arch, and descending aorta: DAo) were drawn for each of the flow datasets (5D flow PT, 5D flow SG, and conventional 4D flow). The velocity streamlines depicted in A correspond to the subject whose respiratory motion had the highest correlation between PT and SG (see Figure 2A), whereas the subject depicted in B is the subject showing the lowest respiratory signal correlation between PT and SG (see Figure 2B). White arrows in the figure denote for each subject 1 location in the aorta where both 5D flow reconstructions show similar streamline patterns while differing from the reference 4D flow. AAo1, aortic root; AAo2, aortic arch; DAo, descending aorta

**FIGURE 5:**
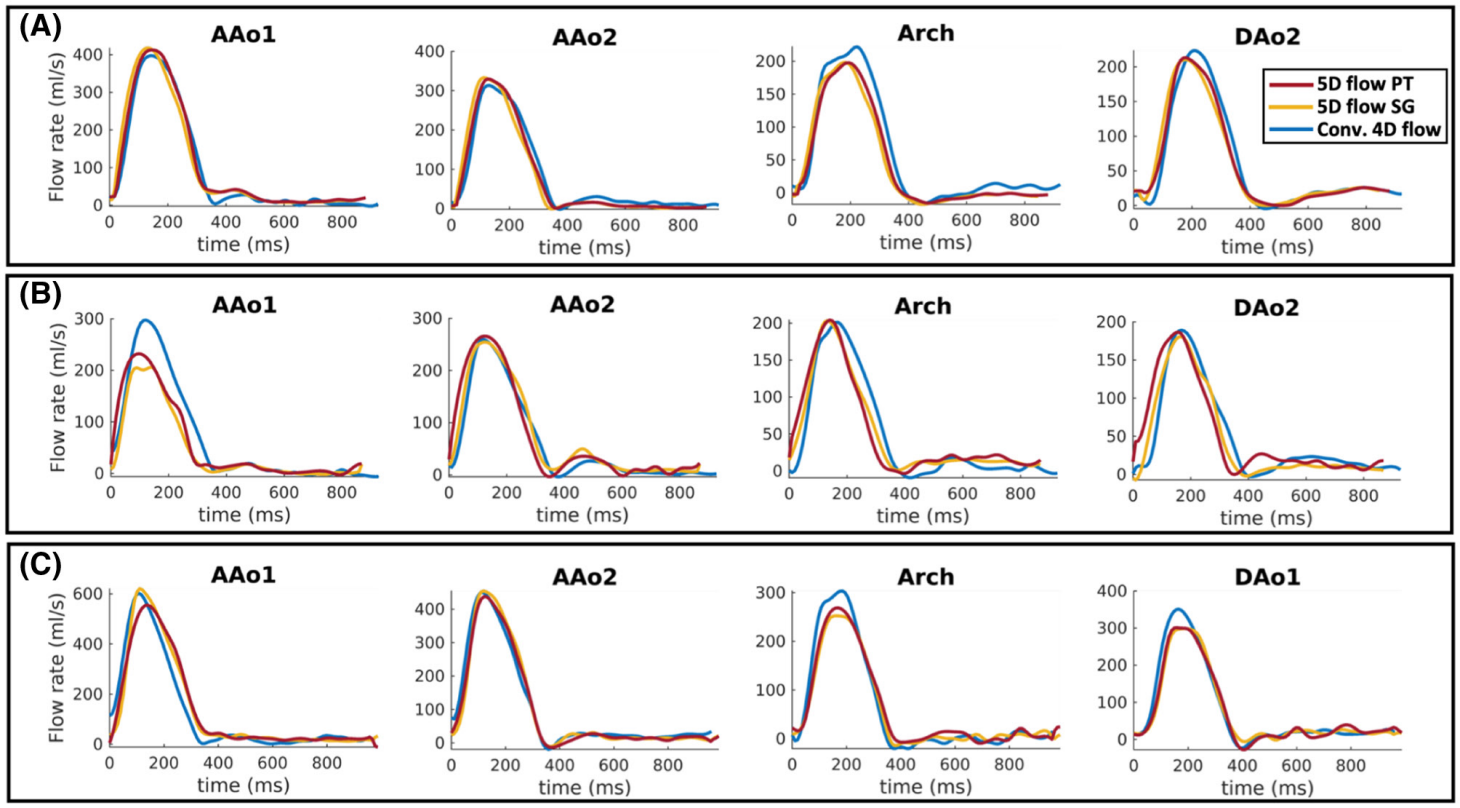
Comparison of aortic blood flow measurements in 3 representative subjects. Flow rate curves for 2 healthy volunteers (A‐B) and 1 patient (C) are shown for 4 regions of interest (ascending aorta: AAo1, ascending aorta pre‐aortic arch: AAo2, end of aortic arch: Arch, and descending aorta: DAo), using 5D flow PT, 5D flow SG, and conventional 4D flow. The flow rate curves depicted in A correspond to the subject whose respiratory motion had the highest correlation between PT and SG (see Figure 2A), whereas the subject depicted in B is the subject showing the lowest respiratory signal correlation between PT and SG (see Figure 2B)

Figure 4 depicts 3D aortic streamlines at peak systole of 2 representative healthy subjects and 1 CHD patient (Marfan syndrome) for the 5D flow PT, 5D flow SG, and 4D flow imaging datasets. A comparison of the magnitude and phase images from 5D flow PT and 5D flow SG reconstructions of a representative subject are included in Supporting Information Figure S2. The 2 healthy subjects selected (Figure 4A,B) correspond to the ones shown in Figure 2A,B (highest and lowest respiratory correlation). Figure 4 also depicts the location of the 2D analysis planes for each case. For each streamline image, white arrows highlight differences between aortic flow streamlines when comparing the two 5D flow and the reference 4D flow datasets. For the 2 healthy subjects, the largest differences between 4D flow streamlines and the remaining ones were reported in the descending aorta, whereas the largest difference reported for the 22‐year‐old CHD patient was at the level of the ascending aorta. Figure 5 shows the flow rate curves of the same subjects using 5D flow PT, 5D flow SG, and 4D flow. In general, the flow rate curves overlap between 5D flow PT and 5D flow SG reconstructions.

**FIGURE 6:**
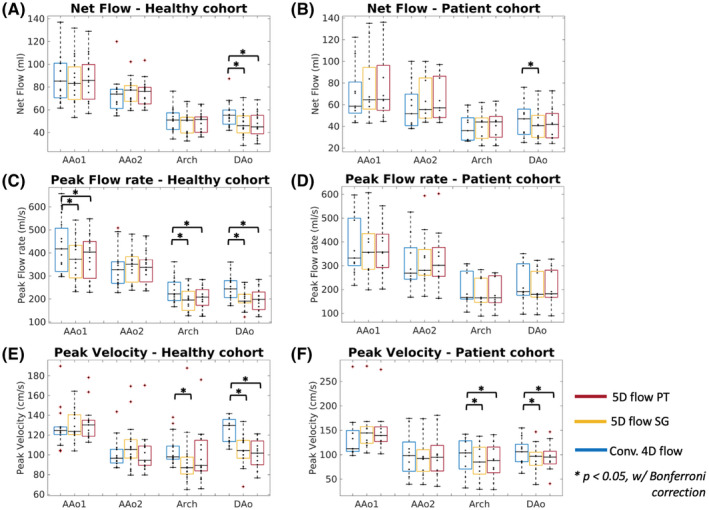
Quantitative evaluation of flow metrics from 15 healthy volunteers and 9 congenital heart disease patients. Net flow (A,B), peak flow rate (C,D), and peak velocity (E,F) measurements from 5D flow PT, 5D flow SG, and 4D flow for the healthy cohort (A,C,E) and patient cohort (B,D,F). No significant differences are reported between the 5D flow PT and 5D flow SG flow measurements for either the healthy or patient cohorts. Both 5D flow PT and 5D flow SG reported significant differences in the flow measurements when compared to 4D flow in both cohorts of the study (main differences in Arch and DAo). Interquartile range is drawn by the box limits; black lines correspond to the sample median; the box whiskers delineate 99.3% coverage assuming a Gaussian distribution of the data; and outliers are marked with a red cross. AAo1: ascending aorta, AAo2: ascending aorta pre‐aortic arch, Arch: end of aortic arch, DAo: descending aorta. *P < .05

The analysis of the different flow measurements across the 2 cohorts (Figure 6) reported similar results to what had already been reported in the flow rate curves for the previous representative cases. In the cohort of healthy subjects, there were no significant differences reported for any of the flow measurements (net flow, peak flow rate, and peak velocity) when comparing the images from 5D flow PT and from 5D flow SG. Conversely, there were some significant differences between 5D flow PT and 4D flow measurements (net flow of DAo, peak flow rate of Arch and DAo, peak velocity of DAo; *P* < .05), and there were also reported significant differences between 5D flow SG and 4D flow measurements (net flow of DAo, peak flow rate of aortic root, Arch and DAo, peak velocity of Arch and DAo; *P* < .05). Likewise, the analysis of the CHD patient cohort showed a good agreement between 5D flow PT and 5D flow SG measurements, and some discrepancies when compared to 4D flow datasets (net flow of DAo for 5D flow SG, and peak velocity of Arch and DAo for both 5D flow reconstructions vs. 4D flow; *P* < .05).

**FIGURE 7:**
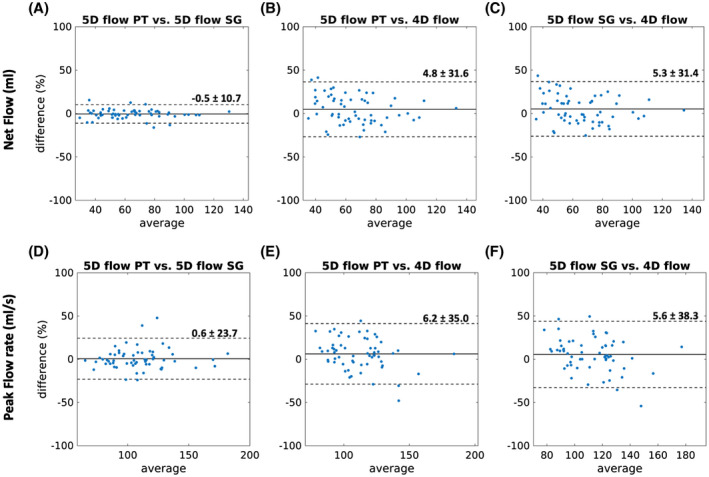
Bland‐Altman plots of average net flow (A‐C) and peak flow rate (D‐F), between 5D flow PT versus 5D flow SG (A,D), 5D flow PT versus 4D flow (B,E), and 5D flow SG versus 4D flow (C,F). Biases reported for each comparison showed a low variability between 5D flow PT and 5D flow SG

Bland‐Altman plots on the healthy cohort analyzing net flow (Figure 7A‐C) reported a bias of −0.5 ± 10.7 ml for 5D flow PT versus 5D flow SG, 4.8 ± 31.6 ml for 5D flow PT versus 4D flow, and 5.3 ± 31.4 ml for 5D flow SG versus 4D flow. Regarding peak flow measurements (Figure 7D‐F), Bland‐Altman plots showed biases of 0.6 ± 23.7 ml/s for 5D flow PT versus 5D flow SG, 6.2 ± 35.0 ml/s for 5D flow PT versus 4D flow, and 5.6 ± 38.3 ml/s for 5D flow SG versus 4D flow. Overall, these results showed good agreement between 5D flow PT and 5D flow SG measurements, and some underestimations relative to the 4D flow reference.

### 3.4 Impact of sequence parameters on PT and SG signals

The average background velocity decreased as the number of readouts acquired per interleave was increased (Table 2). Accordingly, the corresponding decrease in SG sampling frequency led to progressively worse estimations of heartbeat interval duration and trigger jitter. Conversely, the sampling rate of PT remained constant for each configuration of k‐space sampling.

## 4 DISCUSSION

In this study, PT‐based estimation of physiological motion was successfully integrated into the 5D flow framework. With this approach, the extraction of respiratory curve and cardiac triggers, as well as the subsequent flow quantification, were shown to provide equivalent results to the previously described SG framework. Furthermore, we demonstrated both the sensitivity of SG and the insensitivity of PT to changes in the data acquisition, thus highlighting the potential of using PT to further optimize radial 5D flow acquisitions or free‐running whole‐heart imaging in general.

This study details the first use of PT for fully self‐gated whole‐heart imaging, as well as the first comparison between PT and an established SG protocol for both respiratory curve and cardiac trigger extraction. We demonstrated that PT could be successfully applied to both a cohort of healthy individuals and a cohort of CHD patients.

Both PT and SG have been previously individually validated as respiratory‐tracking sources.^14^, ^26^, ^27^ Consequently, we observed a significantly strong positive correlation between PT and SG respiratory curves, as well as no statistically significant differences in liver displacement measurements. Still, the binning distribution variability in the 2 methods suggests there are small differences stemming from the ways in which PT and SG are sensitive to motion. In fact, SG extracts motion from the imaging volume,^26^ and the modulation of the PT is caused by eddy current variations in moving tissues.^17^ Furthermore, the sampling frequencies of the 2 signals are different, as well as the number of data points used for each time sample. The described differences between SG and PT may emphasize some of the disparities obtained after extracting each respiratory curve and cardiac triggers, as discussed below. However, these small differences did not yield any significant quantitative differences for the flow measurements in this work. Furthermore, the reported binning differences appear to be correlated to the range of amplitudes assigned to each respiratory bin, that is, that bins with narrower amplitude ranges (such as end‐expiratory bins) will naturally have fewer overlapping readouts between PT and SG and more matches across the neighboring bins. Therefore, larger binning differences are found in readouts that have similar estimated respiratory amplitudes and as a result cause low motion blur if misplaced.

Prior to comparing the PT and SG cardiac triggers, ECG signal quality assessment revealed a failure to accurately record ECG signals during the 5D flow acquisition in 2 healthy subjects. This may have been caused by inadequate electrode placement, the subject’s physiological features, or by the magnetohydrodynamic effect, as previously reported.^18^, ^28^ Regardless of the cause, this further demonstrates the importance of alternative cardiac gating options for the current 5D flow framework. For the remainder of the subjects, quantitative analysis of cardiac triggers derived from PT and SG showed good agreement with ECG. The heartbeat interval duration measurements showed significant correlation values to ECG, and no significant bias was reported in the trigger jitter measurements between SG versus ECG and PT versus ECG. Additionally, the reported trigger jitter measurements were lower than the temporal resolution; therefore, in the worst‐case scenario, any misplaced bin was only shifted into its neighboring cardiac phase. The results obtained in this comparative analysis clearly show that the integration of PT into the 5D flow sequence presents similar performance to the SG framework. In fact, when looking at the trigger jitter results, we can see a small (and nonsignificant) improvement in jitter measurements for PT when comparing to SG, which could be related to the sharper trigger detection mechanism chosen for PT, as well as to the increased sampling frequency used. Still, the features used for triggering in both the PT and SG pipelines are expected to be less well‐defined than the established R‐wave peak used in ECG triggering. Further understanding of the link between the PT signal and its underlying cardiac physiology may help provide additional improvements to the current framework. Of note, when varying the trajectory architecture setup, PT trigger jitter remained constant relative to decreasing SG performance, which further highlights the potential advantages of PT.

Blood flow measurements derived from 5D flow PT, 5D flow SG, and 4D flow datasets were successfully performed in all 15 healthy subjects and 9 patients with CHD. When comparing 5D flow PT to 5D flow SG, no statistically significant bias was found in their respective net flow and peak flow rate measurements across the 4 examined regions of interest. The peak flow measurements did show a relatively large SD, which may be attributed to the inherent sensitivity to noise relative to the average flow rate. When comparing both methods used for 5D flow image reconstruction to 4D flow datasets, consistent underestimations were observed at the aortic isthmus and the descending aorta. Such discrepancies in flow measurements have been previously reported for 5D flow SG, both in vitro and in vivo.^9^

Phase wraps were reported in a small amount of 5D flow and 4D flow reconstructions, possibly caused by uncorrected aliasing or — in the case of 5D flow datasets — by regularization effects from the compressed sensing reconstruction, as well as possibly the existence of noisy voxels at the edge of the aortic segmentation. However, for a given subject, phase wraps were present in both the PT and SG reconstructions of the same data and therefore did not impact the quantitative comparison of flow measurements.

The radial phyllotaxis sampling trajectory employed in this work has been extensively used for structural and functional imaging^18^ but has only recently been applied to flow‐sensitive imaging.^9^ As such, the sampling scheme that is required to ensure adequate sampling of the SI readout for SG may be adversely affecting the sensitivity to flow by introducing unintended artifacts and effect the background phase. This problem, however, is not unique to our design because other respiratory and cardiac‐resolved 3D anatomical phase contrast protocols implemented by other research groups^8^, ^10^, ^11^, ^29^, ^30^ are also dependent on self‐gating information to extract physiological information and therefore have limited trajectory options for sampling. In this study, we briefly investigated the issue by varying the number of readouts acquired per interleave but keeping the total number of readouts constant (Table 2). This experiment demonstrated, albeit in 1 subject, that by increasing the number of readouts per interleave, we can in fact decrease the background velocity error at the expense of SG cardiac trigger accuracy but without affecting the quality of PT signals. Therefore, PT allows us to decouple the trajectory design from the signal gating methodology and therefore enables the study of different trajectory designs that, for example, would allow for smaller jumps in k‐space and reduce the effect of eddy currents and trajectory related artifacts. Additionally, using the protocol described in this work, the removal of the SI projection required for SG would lead to a ~5% reduction in scan time. Future work should continue to investigate such optimizations and their effect on flow measurements.

The current study was limited by the acquisition time of the conventional 4D flow sequence, which precluded whole‐heart coverage and constrained us to quantitative comparison of flow in the aorta. Nevertheless, the 5D flow framework had already been validated for whole‐heart coverage^9^; thus, our flow analysis on the aorta still provided us with a thorough comparison of PT and SG. Additionally, the lack of an independent ground truth measurement for respiratory motion limited our ability to assess the true accuracy of PT and SG respiratory curves. As a result, we were only able to perform a relative comparison between the 2 signal sources and to evaluate the similarities and differences between them. Finally, an upgrade to the PT system during the course of our study resulted in an increased sampling frequency for the PT signals. This improved system was not available during volunteer and patient scanning but was used to interrogate the impact of sequence parameters on PT and SG signals. Investigating the potential advantages of the increased PT sampling frequency and its impact on respiratory curves, cardiac triggers, and subsequent flow measurements would be of interest for future work.

## 5 CONCLUSION

In this work, the PT navigation system was successfully integrated with the free‐running 5D flow framework as a method for extracting respiratory curves and cardiac triggers to use in the framework’s reconstruction pipeline, providing equivalent results to the previously described self‐gated 5D flow technique in both healthy subjects and CHD patients. Preliminary results also suggest that, in contrast to self‐gating, the PT performance for the extraction of respiratory curves and cardiac triggers may not be affected by the type of radial trajectory chosen for the framework. Therefore, PT may provide new opportunities for trajectory design and sampling schemes in 5D flow MRI with the overarching goal of improving the accuracy of flow measurements in an efficient, predictable, and clinically acceptable scan time.

## CONFLICT OF INTEREST

Dr. Matthias Stuber receives nonmonetary research support from Siemens Healthcare (Erlangen, Germany). Dr. Peter Speier and Mario Bacher are employees of Siemens Healthcare (Erlangen, Germany). The other authors have no relevant conflicts of interest to declare.

## Supporting information


**FIGURE S1** Power spectral density (PSD) of self‐gating and Pilot Tone and the influence of trajectory dependent imperfections. For a set of representative raw self‐gating signals, it is possible to visualize a high‐amplitude frequency component (A.) overlapping with the cardiac frequency range of the signal (0.7‐3Hz). After correcting the signals for trajectory‐related imperfections, the high‐amplitude frequency component disappears from the signal spectrum (B.). Conversely, this peak is not observed in the raw Pilot Tone data (C.), and therefore there is no need for trajectory‐related corrections

**FIGURE S2** Comparison between 5D flow PT and 5D flow SG reconstructions for one sagittal slice in peak systole during end‐expiration. Columns depict (from left to right) the 5D flow PT reconstructed dataset, the 5D flow SG reconstructed dataset and the percent difference between the two datasets. Rows depict (from top to bottom) Magnitude images, velocity images in the y direction, velocity images in the x direction, and velocity images in the z direction

**TABLE S1** List of all congenital heart disease patients included in this study and their corresponding clinical conditions

## Data Availability

All datasets used during the current study, as well as all the code used for the study, are available on request to the corresponding author.
