# Supplementary material for: Pilot tone navigation for respiratory and cardiac motion‐resolved free‐running 5D flow MRI
Source: Magn Reson Med. 2021 Oct 5;87(2):718–32. doi: 10.1002/mrm.29023 (PMC8627452; doi:10.1002/mrm.29023)
Supplement: Supplementary file 3 — TABLE S1 List of all congenital heart disease patients included in this study and their corresponding clinical conditions [file MRM-87-718-s001.docx]

# Supplementary material

| **Subject count** | **Age** | **Gender** | **Diagnosis** |
| --- | --- | --- | --- |
| 1 | 13Y | M | Aortic valve stenosis |
| 2 | 51Y | M | d-Transposition of the great arteries, status-post atrial switch |
| 3 | 55Y | F | Turner syndrome |
| 4 | 33 Y | F | Tetralogy of Fallot, status-post repair with transannular patch |
| 5 | 18Y | M | Bicuspid aortic valve, aortic insufficiency |
| 6 | 13Y | M | Marfan syndrome |
| 7 | 29Y | F | Corctation of the aorta, status-post resection and end-to-end anastomosis |
| 8 | 22Y | M | Marfan syndrome |
| 9 | 16Y | M | Small perimembranous VSD, pulmonary valve stenosis |

**Supporting Information Table S1.** List of all congenital heart disease patients included in this study and their corresponding clinical conditions.


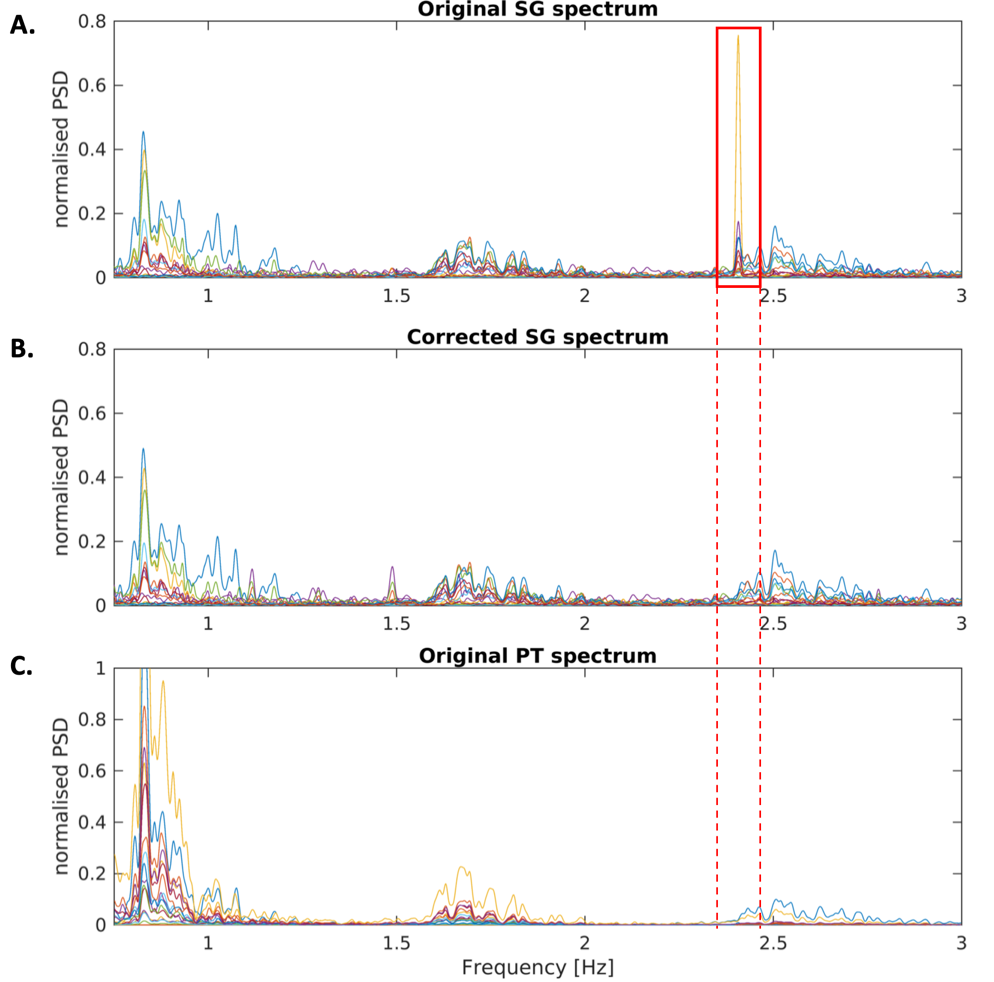


**Supporting Information Figure S1.** Power spectral density (PSD) of self-gating and Pilot Tone and the influence of trajectory dependent imperfections. For a set of representative raw self-gating signals, it is possible to visualize a high-amplitude frequency component (**A.**) overlapping with the cardiac frequency range of the signal (0.7-3Hz). After correcting the signals for trajectory-related imperfections, the high-amplitude frequency component disappears from the signal spectrum (**B.**). Conversely, this peak is not observed in the raw Pilot Tone data (**C.**), and therefore there is no need for trajectory-related corrections.


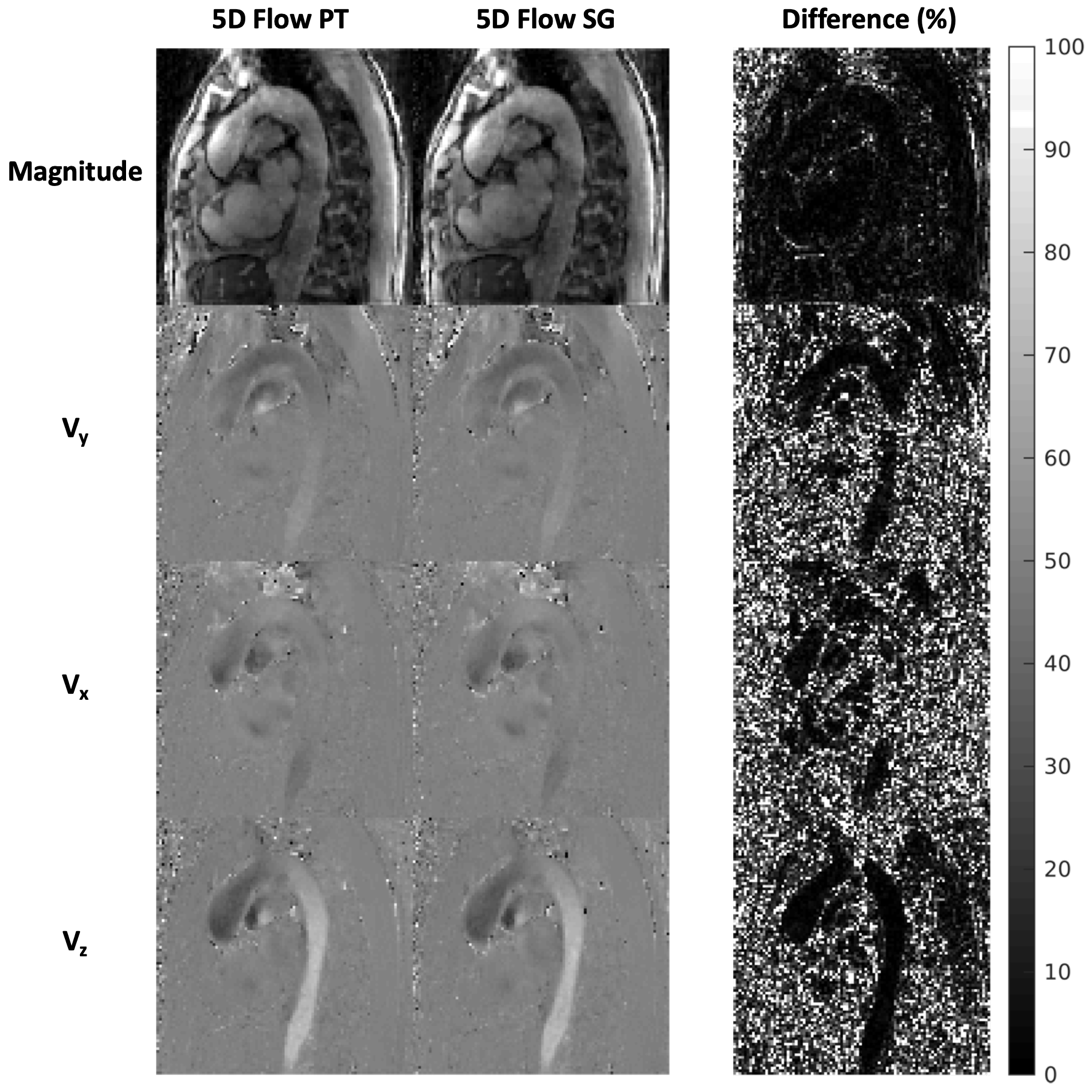


**Supporting Information Figure S2.** Comparison between 5D flow PT and 5D flow SG reconstructions for one sagittal slice in peak systole during end-expiration. Columns depict (from left to right) the 5D flow PT reconstructed dataset, the 5D flow SG reconstructed dataset and the percent difference between the two datasets. Rows depict (from top to bottom) Magnitude images, velocity images in the y direction, velocity images in the x direction, and velocity images in the z direction.
